# Cognitive behavioral therapy-enhanced through videoconferencing for night eating syndrome, binge-eating disorder and comorbid insomnia: a Case Report

**DOI:** 10.1186/s40337-024-01131-8

**Published:** 2024-11-11

**Authors:** Bernou Melisse, Teresa Arora

**Affiliations:** 1 American Center for Psychiatry and Neurology, Al-Manhal, Abu Dhabi, Abu Dhabi, United Arab Emirates; 2 Co-Eur, P.O. box 30514. 3503AH, Utrecht, The Netherlands; 3 Department of Clinical Psychology, Utrecht University, PO Box 80140, Utrecht, 3508 TC The Netherlands; 4 Department of Medical and Clinical Psychology, Tilburg University, Postbus, Tilburg, 90153, 5000 LE The Netherlands; 5 College of Natural and Health Sciences, Department of Psychology, Zayed University, Khalifa City, Abu Dhabi, United Arab Emirates

**Keywords:** Night eating syndrome, binge-eating disorder, Cognitive behavioral therapy-enhanced, Insomnia, Case report

## Abstract

**Background:**

Both night-eating syndrome and binge-eating disorder are characterized by episodes of excessive food consumption, significant distress, and functional impairment related to maladaptive eating behaviors. Both types of eating disorders are associated with poorer sleep quality. Cognitive behavioral therapy has demonstrated good outcomes for binge-eating disorder; however, it is unknown if it is effective for night-eating syndrome and comorbid insomnia.

**Case presentation:**

The current paper presents a case report of a Dutch woman in her 40’s receiving cognitive behavioral therapy-enhanced for night-eating syndrome, as well as binge-eating disorder. However, to tailor the intervention to her specific needs, throughout the course of treatment, cognitive behavioral therapy-insomnia interventions were also implemented. Her comorbid complaints were insomnia, childhood trauma, a depressive mood disorder, and cluster B and C personality traits. She had a history of bariatric surgery, as well as alcohol addiction, and received various treatments in the past, aiming to become abstinent from binge eating.

**Conclusions:**

Post-treatment, she was abstinent from binge eating and, her scores of various eating disorder measures were below clinical cut-points. However, it is unclear if she would show earlier symptom reduction if she received cognitive behavioral therapy-insomnia, prior to cognitive behavioral therapy-enhanced. In addition, the patient reported an increase in her depressive mood and commenced schema therapy after cognitive behavioral therapy- enhanced. Although common, the underlying cause of symptom shifts in patients with an eating disorder remains largely unknown.

**Supplementary Information:**

The online version contains supplementary material available at 10.1186/s40337-024-01131-8.

## Background

Night Eating Syndrome (NES) is defined by the following criteria: evening hyperphagia, in which more than 25% of total daily caloric intake occurs after the evening meal, and/or at least two episodes of nocturnal eating per week, clinically significant distress or impairment resulting from nocturnal eating behaviors and three of the following criteria: morning anorexia, the desire to eat between dinner and sleep, sleep onset insomnia, the belief that food consumption is required to initiate sleep, and evening and nocturnal mood disturbances [1, 2]. NES is formally recognized in the ICD-11, and the International Classification of Sleep Disorders (ICSD-3), but is not included as a diagnostic entity on its own in the DSM-5, rather it is classified as Other Specified Feeding or Eating Disorder [1–4]. Binge-Eating Disorder (BED), defined as the consumption of large amounts of food within a short period while experiencing a loss of control, is formally recognized in both the DSM-5 and the ICD-11 [1, 3]. Both NES and BED involve episodes of excessive food consumption and psychological distress, suggesting significant overlap in clinical presentations [5]. Conversely, differentiating diagnostic criteria between NES and BED are the larger amounts of food eaten, and the loss of control experienced by individuals with BED compared to NES. In addition, individuals with NES consume the majority of food around, or after, dinner [1–3]. Moreover, recent literature reports that individuals with NES experience a loss of control pertaining to food consumption. However, individuals with BED exhibit a greater frequency of binge-eating episodes and display increased levels of overall eating disorder pathology, as compared to individuals with NES [2, 6–8].

The prevalence of NES is estimated to be around 1.5%; however, the prevalence is 15% among individuals with a morbidly high Body Mass Index (BMI; kg/m^2^ > 40), and ranges between 8 and 50% among individuals who have had bariatric surgery [9–11]. In addition, while NES and BED are formally considered mutually exclusive diagnoses [1, 3], approximately 15% of women diagnosed with BED also meet the criteria for NES [9–11]. Furthermore, NES commonly coincides with poorer sleep quality [6, 12], insomnia [13], Post Traumatic Stress Disorder (PTSD), and childhood trauma [14]. Like NES, a significant proportion of individuals with BED also have a high BMI (31%), experience sleep problems (29%), and lifetime PTSD (32%) [12, 15]. Moreover, NES and BED are both associated with poorer quality of life [16, 17] and depressive symptoms [6, 14, 18]. Effective treatments are therefore crucial to reduce the burden of NES and BED.

Relatively little is known about successful psychological interventions for NES. A limited amount of evidence suggests that Cognitive Behavioral Therapy (CBT) may be beneficial [19–21]. However, Cognitive behavioral therapy-enhanced (CBT-E) [22] is a specialized treatment for eating disorders. In addition, CBT-E is one of the recommended evidence-based treatments for BED [23]. At the end of treatment, 37% of the individuals with BED report a full recovery (abstinence from binge eating and score of the eating disorder examination-questionnaire [EDE-Q] below the clinical cutoff) [24]. However, lifetime PTSD predicts a greater frequency of binge-eating episodes at the end of treatment, as well as at follow-up [25]. Moreover, improved sleep duration predicts better treatment outcomes in patients with BED [26].

Given that little is known about the clinical course of NES, and more is known about BED, the present paper presents a case report of a Dutch woman in her 40’s, diagnosed with NES, BED, insomnia, childhood trauma, and indications of cluster B and C personality traits receiving CBT-E for her eating disorders. As far as we know, this is the first study reporting on CBT-E for NES. The present manuscript is prepared in accordance with the CAse REports (CARE) guidelines [27].

## Case presentation

“Rosanne”, a woman in her 40’s, a Dutch citizen, heterosexual, divorced, worked shifts, and had a history of alcohol abuse. Her general practitioner recommended she seek treatment to control disturbed eating after undergoing a gastric bypass. When defining her request for help, “Rosanne” mentioned that she was dissatisfied with her weight and that she wanted to reduce her binge eating frequency. She reported difficulties with maintaining sleep throughout the night, typically waking after one to two hours and then repeatedly waking every hour thereafter. As a result, her total sleep duration was approximately four hours per night, which contributed to her inability to prevent binge eating. She wondered if her binge-eating episodes were due to her diet and stated that she wanted to improve her sleep duration. She also reported that, even when using psychopharmacology (Diazepam), she would still wake up after 30–60 min. Consequently, she reported experiencing disinhibition and poorer cognitive control, which increased the likelihood of her binge-eating episodes.

“Rosanne” suspected that the onset of her eating disorder was related to the sexual abuse she experienced during her childhood by a family member, as well as a consequence of family dynamics. She reported feeling neglected by her mother and characterized her family as “cold”. This emotional detachment led her to seek validation through eating, which provided her with a temporary sense of fulfillment while simultaneously serving as a ‘cry for help’ to express her unmet needs. She recalled frequently requesting porridge/oatmeal or a sandwich from her mother by slipping a note under her bedroom door at night. “Rosanne” was unable to recall exactly when her binge-eating episodes commenced but disclosed that they had been present as long as she could remember. The purpose of her binge-eating episodes, she believed, was to nurture and comfort herself. At approximately eight years old, she was sent to a dietitian for weight loss treatment and consequently received appetite-suppressing medication. When looking back at pictures of her childhood, she struggled to understand why she was referred for weight loss treatment at this young age. She reported, “They made me feel like I was a severely overweight child, but when looking at these pictures I was a bit more curvy compared to my peers, but you can tell that I exercised a lot”. At high school, she commenced purging behaviors and, consequently, she may have developed bulimia nervosa, though no formal assessment was conducted. However, when she was informed about the medical risks of purging behavior, she reduced this to twice a year, to date. Around 15 years before “Rosanne” presented herself for treatment, she underwent bariatric surgery (gastric bypass) in a neighboring country, which resulted in 90 kg of weight loss (from 176 kg to 86 kg). However, her weight loss did not alter her sleep. Subsequently, eight years ago “Rosanne” was in, what she called, “A destructive romantic relationship”. She exhibited intense emotional outbursts, likely driven by her low self-esteem and jealousy, as well as unpredictable episodes of anger. These patterns were also present in other interpersonal relationships. She attributed these behaviors to an insecure attachment developed during childhood, which made it challenging for her to initiate and sustain relationships. Additionally, during the romantic relationship, she developed alcohol dependence, which further exacerbated relational difficulties and led to conflict with one of her daughters.

In January 2024, when she presented herself for treatment, “Rosanne” reported a two-year alcohol abstinence. Based on a face-to-face clinical interview, she was diagnosed with NES, BED, and a depressive mood disorder. For example, she reported experiencing persistent sadness, difficulties with concentration, and pervasive feelings of worthlessness and guilt. In addition, though not formally assessed, cluster B and C personality were also recognized. A few examples were fear of abandonment, feelings of emptiness, difficulties maintaining relationships, and mood swings, as well as the desire for close relationships although she struggled to initiate them, fear of rejection, and sensitivity to negative evaluation. PTSD appeared to be in remission. When she presented for treatment, she had regained approximately 50 kg since the onset of weight regain, which commenced three years post-bariatric surgery and was persistent (current weight: 128 kg, height: 1.72 m, BMI: 43.3). She was receiving weight management treatment with a dietitian and had been engaged in schema therapy for the past six years to address various maladaptive schemas impacting her psychological well-being. Because of intermittent fasting, her daily structure was: 07:00 she started work, at 12:00 she ate a small salad, at 14:00 she had a little snack, e.g. one slice of bread, and at 19:00 a small dinner portion. She reported going to bed at around 21:00–22:00, subsequently waking at around 23:00, and again a few hours later. Several days a week she used to have a binge-eating episode before or after dinner, meeting the criteria of BED. In addition, she had binge-eating episodes at approximately 23:00 and one a few hours later. Consequently, she met the criteria of NES since > 25% of her energy intake was consumed after her evening meal. As a consequence of bariatric surgery, the overall amount she ate during the binge-eating episodes was limited. She commonly ate a fried egg, fruit, porridge/oatmeal, four slices of bread with spread, or yogurt. She used to eat until she was uncomfortably full, and she reported that the purpose of the binge-eating episodes was to nurture and comfort herself [BED]. She was convinced that she needed to feel full to be able to fall asleep [NES]. She stated she could not go to bed without any background noise such as white noise, music, or listening to a podcast.

Though not formally included as a diagnostic criterion of BED in the DSM [1, 28], “Rosanne” reported overvaluation of shape and weight (an excessive significance of shape and weight in determining self-worth), and consequently body checking (looking at the mirror, pinching, squeezing) and avoidance behaviors (avoiding the weight scale, wearing long dresses). In addition, she regularly compared her body shape to others, always when she met someone of a similar age. She had the feeling that people used to treat her differently when she lost weight post-bariatric surgery: “Everybody used to like me. Right now people tend to think I am stupid because I am fat”. Her social support system was limited.

“Rosanne” received various treatments in the past to target abstinence from binge eating. She received Eye Movement Desensitization and Reprocessing (EMDR) which targeted the sexual abuse that she believed had induced binge eating. She had good outcomes concerning PTSD but continued binge eating. She also received individual CBT, group CBT, IPT, eclectic psychotherapy, and non-formal healthcare therapies such as hypnotic therapy, and craniosacral therapy, none of which resulted in binge-eating abstinence.

### Intervention

Governmental settings in the Netherlands are currently dealing with a lack of specialized eating disorder psychologists, resulting in a gap between treatment supply and demand [29]. Consequently, patients with eating disorders experience long waiting periods before they can commence treatment. Therefore, “Rosanne” was referred to a psychologist with a private practice. She contacted the first author, a Dutch female clinical psychologist with a PhD degree specializing in eating disorders, via LinkedIn, following a friend’s recommendation. Though her healthcare insurance would partially cover governmental treatment, “Rosanne’s” employer agreed to reimburse her treatment sessions to prevent absenteeism and presenteeism from work.

During the first session, “Rosanne” was informed that the therapist was not specialized in NES, but that CBT-E displayed superior outcomes for BED over other types of treatments. She was also made aware that CBT was effective for NES. It was emphasized that weight loss was not the primary goal of treatment, given the potential association between dieting and an increased risk of binge eating [30]. In 2015, the therapist completed CBT-E training provided by the Centre for Research on Eating Disorders in Oxford, United Kingdom, and familiarized herself with the detailed CBT-E manual [22], and the book *Overcoming Binge Eating* [31].

**Fig. 1:**
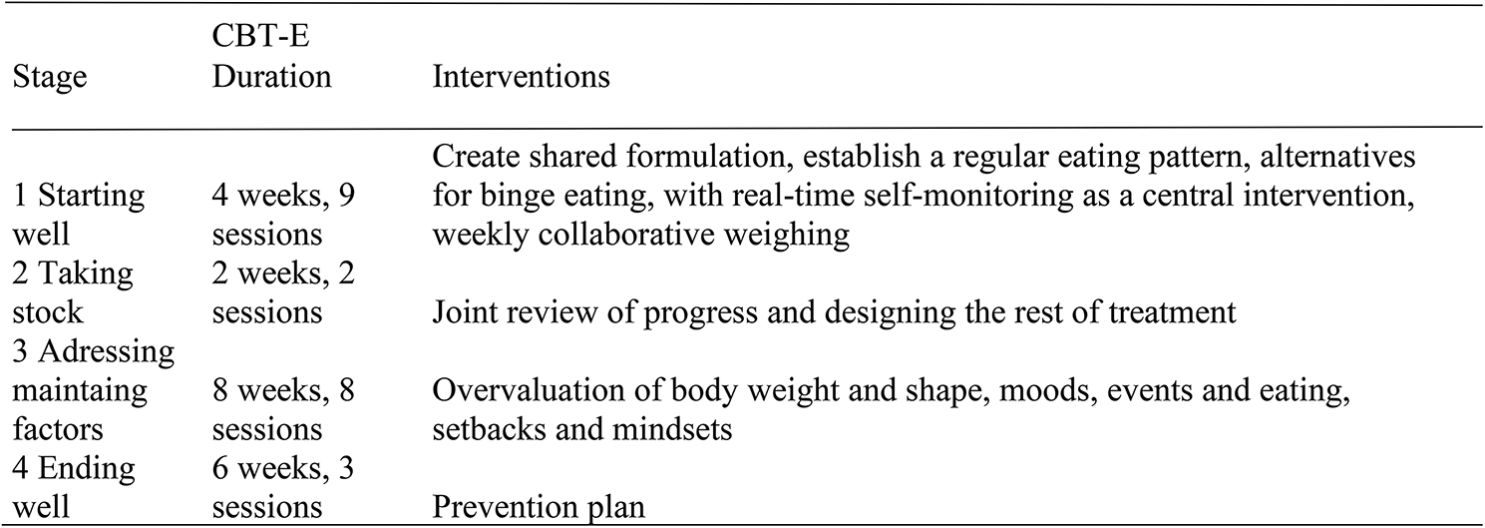
Description of the four stages of CBT-E focused version. CBT-E cognitive behavior therapy-enhanced

Due to the geographical distance between “Rosanne” and the therapist it was decided to conduct treatment through videoconferencing, which has been shown to be effective [32, 33], especially among eating disorder patients with higher BMIs [34]. The patient received individually delivered outpatient CBT-E treatment (focused version), as extensively described in the CBT-E manual [22]. Figure 1 shows that CBT-E consists of four phases. During phase one (starting well; eight sessions over four weeks), a shared treatment formulation was created, a regular eating pattern was established, weekly collaborative weighing was conducted and alternatives for binge eating were offered, with real-time self-monitoring as a central intervention. Subsequently, phase two (taking stock; two sessions over two weeks), entailed a joint review of progress and the remaining treatment was discussed. Stage three (addressing maintaining factors, ten sessions over ten weeks) addressed the overvaluation of body weight and shape as well as moods, events, eating, setbacks, and mindsets. During stage four (ending well, three sessions over six weeks) a relapse prevention plan was created.

CBT-E was merged with some elements of Cognitive Behavioral Therapy for Insomnia (CBT-I) [35]. CBT-I addressed the underlying factors sustaining sleep difficulties. The applied interventions involved sleep restriction: limiting time in bed to the actual sleep duration, and stimulus control: associating the bed only with sleep, and a consistent sleep-wake timing schedule.

### Outcomes

The primary outcome indicator was the reduction in binge-eating episodes at stage two and four compared to pre-CBT-E treatment. The frequency of binge-eating episodes was measured over the previous 28 days using the Eating Disorder Examination (EDE) [36]. The EDE is an extensively used expert interview. The secondary outcome indicator was a reduction in eating disorder pathology below the clinical cutoff of the EDE (EDE global score < 1.77) during stage four, and post-treatment [36, 37]. Outcome measures based on self-reported data included a reduction of eating disorder pathology and body-shape dissatisfaction during the previous 28 days of CBT-E stage four. This was measured using the Eating Disorder Examination-Questionnaire (EDE-Q), which has a clinical cutoff of < 1.79 in the Netherlands [38, 39] and the Body Shape Questionnaire (BSQ), which has a clinical cutoff of < 97 [40, 41]. Both self-report measures exhibit a unidimensional factor structure. The interview assessments, as well as the self-reports, were processed in Qualtrics, which is ISO 27,001/27,002/9001 and NEN 7510 certified [42].

### Ethics

“Rosanne” was informed about the present paper, and signed an informed consent form, in accordance with the World Medical Association Declaration of Helsinki (World Medical Association, 2001). According to the Dutch Central Ethical Commission on Medical Research with Human Subjects (Dutch abbreviation: CCMO), analysis of anonymized routine outcome monitoring data does not require additional approval from a local medical-ethical approval board.

### Course of treatment and complicating factors

CBT-E was provided as per the protocol [22]. Upon starting treatment, she discontinued intermittent fasting, as this eating pattern has been associated with an increased risk of binge- eating episodes in some individuals [43]. To prevent “Rosanne” from malnourishment, due to bariatric surgery, she agreed to consult a clinical dietitian who specialized in bariatric surgery and eating disorders during her treatment. She was advised to slightly increase her protein intake. Furthermore, several patterns emerged from her food-monitoring records: “Rosanne” only ate when she felt hungry, reported feeling ‘fat’ while eating, expressed dissatisfaction with the foods she consumed, and avoided eating in the presence of others. During week two of treatment (stage one), when eating regularly was introduced, “Rosanne” commenced having breakfast and morning snacks. Consequently, she noticed a significant reduction in the frequency of binge eating. However, since “Rosanne” continued her binge-eating episodes at night, some specific adjustments were made to tailor the treatment towards her specific needs. It was agreed that she would not eat in bed anymore, neither when having breakfast in bed during the weekends nor when her granddaughter slept over, which was common. She would only eat at the dinner table in the kitchen, in an attempt to weaken the bed-food association she had developed. She noticed that when she used Diazepam, which was prescribed to use as needed (5–10 milligrams), the likelihood of bingeing at night increased. When she experimented by increasing the dose (10 milligrams), the likelihood of binge eating increased even more as it led to poorer cognitive control, including disinhibition. She therefore agreed, temporarily, to reduce her intake of Diazepam. Since “Rosanne” was convinced that she needed to have a full stomach to fall asleep, she used to have food on her bedside table. To reduce the temptation of binge eating, she agreed to remove the food. When going to the bathroom at night she developed a habit of eating, therefore it was suggested to replace eating with sips of water. This led to a reduction in the frequency of binge eating, however, she still found herself going to the kitchen at night. To minimize this, she placed a wet towel in front of her kitchen door to create a barrier to binge and disrupt this automatic behavior.

As a consequence of her increased awareness through discussion during the sessions, the frequency of nocturnal binge eating decreased but another issue emerged. She was awake for hours, being unable to sleep and, though remaining abstinent, her urge to consume alcohol increased. Following the CBT-E protocol [22] she monitored the duration of the urges to binge. She made a list of alternatives for binge eating including things that would not wake her up too much, e.g. reading a book, crochet, etc. However, her insomnia symptoms continued to interfere with her treatment. Despite it not being common practice to merge evidence-based treatment protocols in the Netherlands, and the therapist did not possess specialized training in insomnia, the decision was made to integrate urge-surfing with elements of CBT-I [35]. This decision could be justified by distinguishing between eclectic treatments and well-informed, research-supported modifications. Furthermore, this approach was consistent with Fairburn’s recommendation that therapists individualize case formulations and incorporate additional therapeutic modules, as needed [22, 44]. The tailored recommendations [45, 46] were as follows: (i) only going to bed when sleepy; (ii) avoid using the bed for other activities than sleeping and, if applicable, for sexual intercourse; (iii) get out of bed if wakefulness persists for more than 15–20 min; (iv) get up at the same time every day. Consequently, her sleep efficiency improved, and binge eating at night was reduced.

During stage three of treatment [22], she learned strategies to realize having a binge was a choice instead of something she could not control. Addressing event-related changes in eating was applied. It was essential to identify potential eating-related challenges as early as possible, she then explored potential solutions, and evaluated their positive and negative outcomes. If she voluntarily decided to have a binge, she was only allowed to do so at the kitchen table. In addition, alternative coping mechanisms were addressed by targeting mood intolerance and binge analysis. Through binge analyses, she discovered that she was at a heightened risk of binge eating when more than 2.5 h elapsed between meals, and when she violated a dietary rule.

**Fig. 2:**
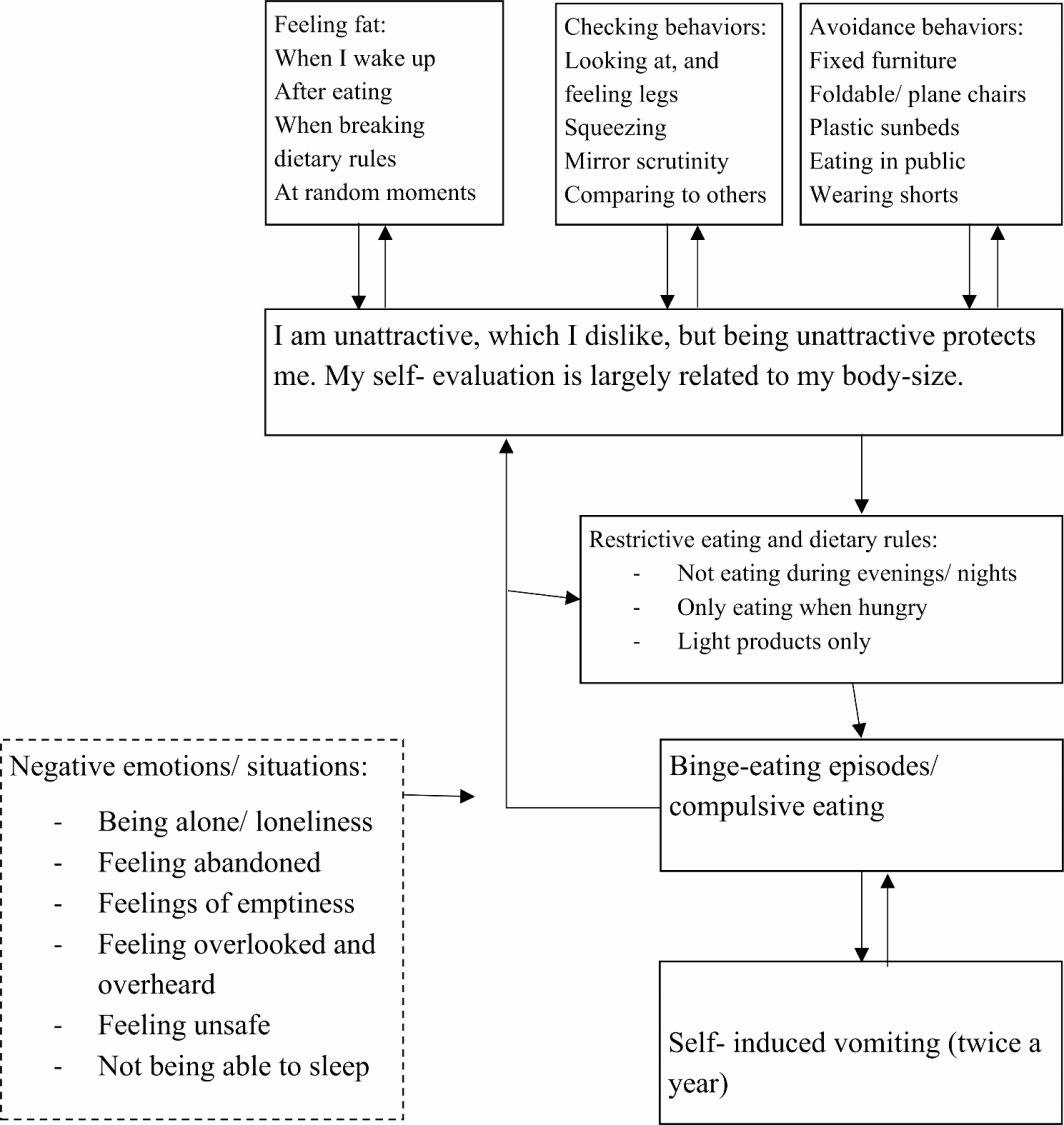
Cognitive Behavior Therapy-Enhanced case conceptualization including the extended formulation

The overvaluation of shape and weight was also addressed during stage three. She recognized that her self-evaluation was primarily based on shape and weight, accounting for over 80% of her self-assessment. It was recommended that she engage in activities unrelated to appearance, which led her to start arts and crafts and join a choir. However, a male choir member frequently commented on her body. In treatment, the therapist practiced with her how to address this situation. She monitored her body-checking behaviors, including mirror scrutiny and stomach squeezing. She also avoided eating in public and using certain furniture (Fig. 2). Gradually, she worked on minimizing body-checking behaviors and, when these occurred, she was instructed to challenge the associated thoughts. She then commenced eating in public, stopped avoiding certain types of furniture, and compared her body to a diverse range of individuals at the supermarket, rather than focusing on thin individuals. However, due to the severity of the overvaluation, it was deemed not possible to reduce the overvaluation to a satisfactory level. Instead, it was suggested that she would continue the strategies that were offered, post-treatment. Conversely, binge eating functioned as a mechanism for suppressing negative emotions. As binge eating decreased, there was a corresponding reduction in rewarding experiences, which potentially led to a literal sense of emptiness, and subsequently affected her mood negatively. As part of her relapse prevention plan, it was recommended that “Rosanne” continue elsewhere, to receive schema therapy and to address her depressive mood. After treatment concluded, she commenced schema therapy with a specialized therapist.

### Treatment outcomes

Table 1 shows that the frequency of binge-eating episodes, the severity of eating disorder pathology, and the severity of body-shape dissatisfaction, reduced between the start of treatment and phase two, five weeks after CBT-E commenced. At post-intervention, she was abstinent from binge eating and scored below the clinical cutoff for all measures. The only exception was for the EDE-Q, which potentially stemmed from her levels of shape and weight concern. ”Rosanne” fully recovered from NES and BED. During the last 28 days of treatment, she did not have any nocturnal eating episodes, was abstinent from binge eating and her EDE score was below the clinical cutoff. Her BMI remained stable throughout the course of treatment.

**Table 1:** Changes in number of binge eating episodes, EDE, EDE-Q and BSQ scores over the course of treatment

	Time of measurement		
	Pre-intervention, *M*	Phase 2, *M*	Post-intervention, *M*
Number of objective binge-eating episodes ^a^	24	4	0
EDE global score ^a^	2.9	2.25	1.4
EDE shape concern ^a^	4.3	3.8	3.3
EDE weight concern ^a^	3.8	3.2	2.2
EDE eating concern ^a^	1.8	1.4	0
EDE dietary restraint ^a^	1.8	0.6	0.2
EDE-Q ^a^	3.6	3.3	3.3
BSQ ^a^	108	94	89

^a^ Measured during the past 28 days

M = mean, EDE = eating disorder examination, EDE-Q = eating disorder examination- questionnaire, BSQ = body shape questionnaire.

Follow-up measures were not administered as “Rosanne” continued with schema therapy elsewhere. Maintaining contact was deemed undesirable for the effectiveness of the schema therapy. Additionally, measuring treatment outcomes would have been unclear in terms of which effect was being assessed.

## Discussion and conclusions

Based on EDE interview data, which are considered more reliable compared to self-reports [47], “Rosanne” fully recovered from NES and BED. She was abstinent from nocturnal eating and binge eating. The severity of eating disorder pathology, and body-shape dissatisfaction, were below the clinical cutoffs. However, she self-reported clinical levels of eating disorder pathology. Various studies have indicated a discrepancy between interview data and self-reports, stemming from a lack of knowledge and socially desirable behaviors [47–49]. When discussing this discrepancy with her, she addressed that though she was aware that weight loss was not a goal of treatment, she was disappointed that she did not lose weight. Consequently, her shape and weight concerns increased. Following what was addressed during treatment, it was discussed that she first had to stabilize her weight. To achieve this, abstinence from binge eating was the primary goal [50]. In addition, the results of the present case report were not in line with a study that showed that childhood trauma predicts poorer treatment outcomes [14]. Though no assessment tools were administered to determine depressive mood during treatment, she reported experiencing increased depressive mood. The disappearance of a maladaptive coping strategy, specifically binge eating, potentially resulted in the emergence of other psychological symptoms, or the development of alternative maladaptive coping mechanisms. Symptom shifts were commonly observed during her eating disorder treatments. However, the underlying processes driving these shifts remained largely unknown [51].

Consistent with the findings of Allison et al. (2010), who investigated the efficacy of CBT-BED, a precursor of CBT-E, in patients with NES [19], “Rosanne” demonstrated significant improvement following CBT-E and no longer met the diagnostic criteria for NES or BED, post-treatment. She also reported insomnia symptom improvement. However, the therapist wondered if “Rosanne” would have exhibited improvement earlier if CBT-I [35] had been conducted before commencing [35] had been conducted before commencing [22]. This is particularly important given that early change predicts better treatment outcomes at follow-up [52]. However, when she presented for treatment, her primary complaint was to reduce binge eating. Therefore, it was decided to conduct CBT-E. In addition, it was not confirmed if binge eating caused insomnia or if insomnia caused binge eating. The therapist believed that insomnia symptoms would improve if she would had fewer binge-eating episodes throughout the day since binge eating is associated with poorer sleep quality [12]. Vice versa, being able to maintain nocturnal sleep could potentially increase cognitive control and result in a reduction of binge-eating episodes. Moreover, a potential limitation of this case report was the integration of CBT-E with selected elements of CBT-I to individualize “Rosanne’s” treatment, according to her specific needs. Consequently, it was not feasible to disentangle the effects of the individual interventions and, as a result, only the combined outcome of the integrated therapeutic approach could be evaluated. Another limitation of the present study was the lack of standardized instruments to assess insomnia and depression levels. Moreover, no formal measures were employed to evaluate treatment acceptability and satisfaction. These factors were discussed verbally with the patient during the evaluation (phase two), and phase four of treatment. The patient reported being satisfied with the treatment and expressed a desire to have received it 40 years earlier. Finally, since “Rosanne” commenced schema therapy elsewhere, the long-term results, post-CBT-E could not be determined.

In conclusion, this case report shows that CBT-E could be an effective treatment for NES combined with BED. Future studies, however, are recommended to examine if CBT-E should be combined with CBT-I, and, if so, in which order, so that the best approach for patient outcomes can be determined. Increased knowledge about the underlying processes in symptom shift among eating disorder treatment can potentially enhance treatment outcomes concerning comorbid psychiatric complaints.

## Electronic supplementary material

Below is the link to the electronic supplementary material.

Supplementary Material 1

## Data Availability

No datasets were generated or analysed during the current study.

## Abbreviations

BED: Binge-Eating Disorder
BMI: Body Mass Index
CBT-E: Cognitive Behavioral Therapy- Enhanced
NES: Night Eating Syndrome
