# Supplementary material for: Cognitive behavioral therapy-enhanced through videoconferencing for night eating syndrome, binge-eating disorder and comorbid insomnia: a Case Report
Source: J Eat Disord. 2024 Nov 11;12:175. doi: 10.1186/s40337-024-01131-8 (PMC11556170; doi:10.1186/s40337-024-01131-8)
Supplement: Supplementary file 1 — Supplementary Material 1 [file 40337_2024_1131_MOESM1_ESM.pdf]

CARE case report guidelines

# CARE Checklist of information to include when writing a case report

(cc) BY-NC-ND

| Topic | Item | Checklist item description | Reported on Line |
|---|---|---|---|
| Title | 1 | The diagnosis or intervention of primary focus followed by the words "case report" | p1 line 1 |
| Key Words | 2 | 2 to 5 key words that identify diagnoses or interventions in this case report, including "case report" | p2 line 18 |
| Abstract (no references) | 3a | Introduction: What is unique about this case and what does it add to the scientific literature? | p2 line 6 |
| | 3b | Main symptoms and/or important clinical findings | p2 line 7-11 |
| | 3c | The main diagnoses, therapeutic interventions, and outcomes | p2 line 7-11 |
| | 3d | Conclusion—What is the main "take-away" lesson(s) from this case? | p2 line 15-19 |
| Introduction | 4 | One or two paragraphs summarizing why this case is unique (**may include references**) | p5 line 13 to page 6 line 23 |
| Patient Information | 5a | De-identified patient specific information. | page 7 |
| | 5b | Primary concerns and symptoms of the patient. | page 6 |
| | 5c | Medical, family, and psycho-social history including relevant genetic information | page 6 and 7 |
| | 5d | Relevant past interventions with outcomes | page 8 line 1-6 |
| Clinical Findings | 6 | Describe significant physical examination (PE) and important clinical findings. | NA |
| Timeline | 7 | Historical and current information from this episode of care organized as a timeline | page 9 line 3-15 |
| Diagnostic Assessment | 8a | Diagnostic testing (such as PE, laboratory testing, imaging, surveys). | page 9 line 17-24 page 1 |
| | 8b | Diagnostic challenges (such as access to testing, financial, or cultural) | page 8 line 9-17 |
| | 8c | Diagnosis (including other diagnoses considered) | page 7 line 1-2 |
| | 8d | Prognosis (such as staging in oncology) where applicable | NA |
| Therapeutic Intervention | 9a | Types of therapeutic intervention (such as pharmacologic, surgical, preventive, self-care) | page 8 line 8-23, page 9 |
| | 9b | Administration of therapeutic intervention (such as dosage, strength, duration) | page 8 line 8-23, page 9 lin |
| | 9c | Changes in therapeutic intervention (with rationale) | page 11, page 12 line 1-12 |
| Follow-up and Outcomes | 10a | Clinician and patient-assessed outcomes (if available) | page 12 line 14-23, |
| | 10b | Important follow-up diagnostic and other test results | page 12 line 22, 23 |
| | 10c | Intervention adherence and tolerability (How was this assessed?) | NA |
| | 10d | Adverse and unanticipated events | page 12 line 10 |
| Discussion | 11a | A scientific discussion of the strengths AND limitations associated with this case report | page 14 line 13 |
| | 11b | Discussion of the relevant medical literature **with references**. | page 13 and 14 |
| | 11c | The scientific rationale for any conclusions (including assessment of possible causes) | page 13 line 8-23 |
| | 11d | The primary "take-away" lessons of this case report (without references) in a one paragraph conclusion | page 14 line 17 |
| Patient Perspective | 12 | The patient should share their perspective in one to two paragraphs on the treatment(s) they received | page 15 line 13 |
| Informed Consent | 13 | Did the patient give informed consent? Please provide if requested | Yes ☒ No ☐ |
